# Investigation of the clinical utility of adhesion molecules in the management of thyroid nodules

**DOI:** 10.1038/s41598-023-31302-7

**Published:** 2023-03-11

**Authors:** Larissa Teodoro Rabi, Karina Colombera Peres, Matheus Nascimento, Elisangela de Souza Teixeira, Leandro Luiz Lopes de Freitas, Icléia Siqueira Barreto, Murilo Vieira Geraldo, Lígia Vera Montalli Assumpção, Valdemar Máximo, Alfio José Tincani, Natassia Elena Bufalo, Laura Sterian Ward

**Affiliations:** 1 Laboratory of Cancer Molecular Genetics, School of Medical Sciences (FCM), University of Campinas (UNICAMP), Campinas, SP Brazil; 2 Department of Biomedicine, Nossa Senhora do Patrocínio University Center (CEUNSP), Itu, SP Brazil; 3 Institute of Health Sciences, Paulista University (UNIP), Campinas, SP Brazil; 4 Department of Medicine, Max Planck University Center, Indaiatuba, SP Brazil; 5 Department of Pathology, School of Medical Sciences, University of Campinas (UNICAMP), Campinas, SP Brazil; 6 Department of Medicine, São Leopoldo Mandic and Research Center, Campinas, SP Brazil; 7 Laboratory of RNA Biology, Institute of Biology, University of Campinas (UNICAMP), Campinas, SP Brazil; 8 Division of Endocrinology, Department of Medicine, School of Medical Sciences (FCM), University of Campinas (UNICAMP), Campinas, SP Brazil; 9 Department of Pathology, Faculty of Medicine, University of Porto, Porto, Portugal; 10 Institute for Research and Innovation in Health (i3S), University of Porto, Porto, Portugal; 11 TMB Clinic, Campinas, SP Brazil; 12 Head and Neck Surgeon, Surgical Department, University of Campinas, Campinas, SP Brazil

**Keywords:** Cancer, Thyroid cancer

## Abstract

To better understand the relationship among cell adhesion molecules (CAM) and investigate the clinical diagnostic and prognostic application of ICAM-1 (*ICAM1*), LFA-1 (*ITGAL*), and L-selectin (*SELL*) proteins and mRNA corresponding expression in thyroid cancer. Gene expression was evaluated by RT–qPCR, and protein expression was evaluated by immunohistochemistry. We evaluated 275 patients (218 women, 57 men, 48.4 ± 14.5 years old), including 102 benign and 173 malignant nodules. The 143 papillary thyroid carcinoma (PTC) and 30 follicular thyroid carcinoma (FTC) patients were managed according to current guidelines and followed-up for 78.7 ± 54.2 months. Malignant and benign nodules differed concerning mRNA (*p* = 0.0027) and protein (*p* = 0.0020 for nuclear) expression of L-selectin and ICAM-1 (mRNA: *p* = 0.0001 and protein: *p* = 0.0014) and protein expression of LFA-1 (*p* = 0.0168), but not mRNA expression of LFA-1 (*p* = 0.2131). *SELL* expression was more intense in malignant tumors (*p* = 0.0027). *ICAM1* (*p* = 0.0064) and *ITGAL* (*p* = 0.0244) mRNA expression was higher in tumors with lymphocyte infiltrate. ICAM-1 expression correlated with younger age at diagnosis (*p* = 0.0312) and smaller tumor size (*p* = 0.0443). Also, LFA-1 expression correlated with higher age at diagnosis (*p* = 0.0376) and was more intense at stage III and IV (*p* = 0.0077). In general, the protein expression of the 3 CAM decreased as the process of cellular dedifferentiation occurred. We suggest that the *SELL* and *ICAM1* genes and L-selectin and LFA-1 protein expression may help confirm malignancy and assist in the histological characterization of follicular patterned lesions, but we were unable to correlate these CAMs with patient outcomes.

## Introduction

Cell adhesion molecules (CAMs) are glycoproteins present in the cell membrane^1^. They play an important role in inflammatory and neoplastic diseases by recruiting immune cells to injured sites^2,3^. The migration of cells from the immune system consists of four main steps: capture, rolling and activation, arrest, and transmigration^2,3^. Initially, leukocytes are attracted to the vascular endothelium mainly by selectin molecules. Rolling begins with contact with chemoattractant and leukocyte activation. After that, integrins interact with immunoglobulins, enabling adhesion to the endothelial surface and transmigration^4^.

L-selectin, ICAM-1 and LFA-1 are pivotal CAMs that collaborate in the cell migration process from capture to the transmigration process but acting at different stages of the process and in different ways. The literature has been reporting their expression and potential role in aggressive cancers, but the way they interact in the progression and proliferation of thyroid cells is still unclear.

L-selectin is a transmembrane molecule encoded by the *SELL* gene. Its cleavage site allows a soluble form of the molecule, and the cleavage of the structure occurs after its activation^5,6^. L-selectin expression was initially considered exclusive to the leukocyte surface^3,5^, but other cell types and several types of cancer also showed this molecule^7–10^. In general, L-selectin favors interactions that allow both leukocytes^3^ and metastatic tumor cells^7–10^ to start the rolling process. L-selectin expression in thyroid neoplastic cells has been related to more aggressive tumor behavior since it was found to be associated with lymph node metastasis^7^ and BRAFV600E mutation^11^.

ICAM-1 is a transmembrane glycoprotein generally expressed on the surface of several cell types, such as leukocytes, endothelial cells, and fibroblasts^12^. This protein is involved in the regulation of leukocyte trafficking across the endothelial barrier and therefore plays a key role during the inflammatory response. ICAM-1 interacts with lymphocyte function-associated antigen 1 (LFA-1), facilitating the migration of immune cells to the injured site^12^. This binding promotes stable adhesion of leukocytes to endothelial cells, which is an essential step for recognition of the transmigration site. Studies have found that ICAM-1 is overexpressed in different types of cancer^13^, including thyroid cancer, and has associated it with aggressive features such as extrathyroidal extension, lymph node metastasis, and BRAFV600E mutation^14–16^.

LFA-1 is an integrin also known as ALB2 integrin and is encoded by the *ITGAL* gene. Only leukocytes express the B2 group integrins^17^, which are inactive under basal conditions. However, when chemoattracted to injury or tumor sites, they experience conformational changes that expose allosteric sites and allow interaction with other CAMs^18^. The main ligand of LFA-1 is ICAM-1^19^, which binds with high affinity^20,21^ and acts in the stable adhesion step^22^. This molecule is expressed on the surface of all leukocytes^23^ and has recently been described in several types of tumor cells, including breast^24^, colorectal^25,26^, and melanoma^20^. LFA-1 expression has been associated with metastatic progression^10^, whereas the B2 integrin expression decrease in colorectal cancer tumor cells has been correlated with lower chances of liver metastasis outbreaks^27^.

This study focuses on L-selectin, ICAM-1 and LFA-1 to better understand their role in the migration of neoplastic cells seeking possible practical applications in the diagnosis and prognosis of thyroid cancer.

## Materials and methods

This study was conducted at the Laboratory of Molecular Genetics of Cancer (GEMOCA) and approved by the Faculty of Medical Science of State University of Campinas Research Ethics Committee (IRB00002737 and CAAE#65254916.9.0000.5404) and all methods were performed in accordance with the relevant guidelines and regulations. Informed consent was obtained from all individual participants included in the study.

### Patients

We evaluated a total of 275 thyroid nodule paraffin blocks from 218 women and 57 men, 48.4 ± 14.5 years old, patients referred to the Clinical Hospital FCM/UNICAMP and the TMB Private Surgical Clinic in Campinas/São Paulo/Brazil, who underwent partial or total thyroidectomy. Of those, 102 (37%) nodules were benign (48 goiters and 54 follicular adenomas–FA), and 173 (63%) were malignant (143 papillary thyroid carcinomas-PTC and 30 follicular thyroid carcinomas-FTC). The PTC included 55 classic PTC (CVPTC). Since we aimed to better understand CAMs role in aggressive tumors, our cohort was enriched with 38 follicular variants of PTC (FVPTC), 27 oxyphilic variants of papillary thyroid carcinoma/Hürthle cell carcinoma (OVPTC), and 23 tall cell variants of PTC (TCPTC). Hurthle cell tumors were defined as those that presented more than 75% of the tumor cells with oncocytic characteristics^28^. Most of the 173 malignant samples (n = 123, 71.1%) were from patients younger than 55 years. Most patients in this group (n = 44, 77.2%) were TNM I and TNM II (n = 13, 22.8%).

All patients were managed according to a well-established protocol in accordance with international consensuses and followed-up for 78.7 ± 54.2 months^29,30^. Data were collected from medical records and included sociodemographic information, use of medications, and performed tests, including ultrasound, whole body scan, X-rays and other imaging tests, serum TSH, thyroglobulin and other eventual serum measurements, presurgical clinical data, surgery description, and anatomopathological tissue examination. Diagnostic aggressiveness was determined according to the TNM system of classification and staging of differentiated thyroid carcinoma (DTC). A disease-free status was defined as the absence of detectable residual disease (on ultrasound and WBS) and low basal (< 0.2 ng/mL) Tg serum levels. Persistent disease was defined as the presence of a detectable residual or metastatic tumor on imaging methods and/or elevated basal (> 0.2 ng/mL) Tg serum levels. Tumor recurrence was defined as structural disease diagnosed more than one year after ablation in patients without persistent disease. From the total, 57 patients evolved free of disease, 69 patients presented lymph node and/or distant metastasis, and 7 patients died due to DTC during the observation period of this study. The remaining patients could not be included with certainty in any of these groups and were excluded from further statistical evaluation concerning evolution or prognosis. All tissues were examined by two experienced pathologists with particular attention to tumor extension beyond the thyroid capsule (extrathyroidal extension), angiolymphatic, vascular and/or perineural invasion (invasion) and lymphocytic infiltration.

Unfortunately, we could not extract good quality mRNA from all paraffin blocks, but we were able to further investigate mRNA expression in 191 thyroid nodule patients (149 women and 42 men, 47.5 ± 14.4 years old) comprising 97 benign nodules (47 goiters and 50 FA) and 94 malignant nodules (74 PTC and 20 FTC). The PTC included 29 CVPTC, 21 FVPTC, 12 OVPTC and 12 TCPTC cases.

We also obtained 3 normal thyroid tissues from the necropsy of patients who had no history or signs of thyroid disease that were used as control samples.

We excluded patients with a history of accidental or medical exposure to ionizing radiation, other malignancies, autoimmune diseases, and pregnant women and children under 18 years old. FVPTCs suspected of being noninvasive follicular thyroid neoplasms with papillary-like nuclear features (NIFTPs) were excluded from this study.

### Immunohistochemistry analysis

A tissue microarray (TMA) was assembled from formalin-fixed paraffin-embedded tissues representative of tumor regions, previously selected by two experienced pathologists. Each case was added to the TMA in duplicate. Five micrometer sections were cut with the microtome CUT5062 (SLEE Medical, Mainz, DE). The immunohistochemistry reaction was carried out with deparaffinization and rehydration of the tissues. Endogenous peroxidase was blocked with 10 volumes of hydrogen peroxide. Tissues were incubated in Trizma-Ethylenediaminetetra acetic Acid (Tris–EDTA) buffer antigen recovery buffer for 40 min at 97 °C in a steamer (Hamilton Beach Brands, Virginia, USA) and left to rest in 3% milk for 30 min. We added 150 µL of antibody to each slide using specific antibodies for each protein (L-selectin—Anti-CD62L, polyclonal antibody, dilution 1:50; ICAM-1—Anti-ICAM1, monoclonal antibody, dilution 1:1000 and LFA-1—Anti-CD11a, monoclonal antibody, dilution 1:750). The antibody dilution tests were previously performed individually with their respective positive controls. The slides were then incubated in a humid chamber (60 min at 37 °C) and stored in a refrigerator (4 °C) for 16–18 h. Slides were washed in Phosphate-buffered Saline (PBS) 1X, 150 µL of secondary antibody was added and they were incubated (30 min at 37ºC). After that, 3.3-diaminobenzidine-tetrahydrochloride (DAB—150 µL) was added onto the slides before they were packed in PBS 1X until counterstaining with Mayer’s hematoxylin. Positive and negative controls were run in the same batch of reaction. Different concentrations of alcohol and xylol were used to dehydrate the tissue The final score was assigned based on slide analysis performed independently by two different pathologists with extensive thyroid experience, both blinded to tumor characteristics. The analysis included an evaluation of the intensity (weak, moderate, and strong) and the proportion (focal or diffuse) of expression of the studied proteins.

### RNA extraction and reverse transcription

Total RNA from 191 formalin-fixed paraffin-embedded (FFPE) thyroid tumor samples was extracted using the Recover All* Total Nucleic Acid Isolation Kit FFPE (Life Technologies, California, USA) according to the manufacturer’s instructions. All samples were treated with DNaseI (Thermo Fisher Scientific, Waltham, EUA). RNA purity was confirmed by spectrophotometry using the Epoch™ Spectrophotometer System (BioTek Instruments Inc., Vermont, USA) with the Take3™ Multivolume Plate.

First-strand complementary DNA (cDNA) synthesis was performed using the High-Capacity cDNA Reverse Transcription Kit (Life Technologies, California, USA) according to the manufacturer’s instructions. The initial RNA template used for the reaction was 1000 ng. All samples were amplified in a thermocycler (Biosystems, Barcelona, ES) according to the manufacturer’s instructions. After the reaction, the cDNA was diluted to a final concentration of 5 ng/µL.

### Primer optimization and reference gene testing

Primers were designed and verified using the free online software Primer3Plus^31^, OligoAnalyzer^32^, and *insilico*PCR^33^ and were tested at concentrations of 100 nM, 200 nM, 400 nM, and 800 nM, evaluating the best concentration without losses in the amplification cycle due to the absence of primers in the reaction. We used the following primers and concentrations for the respective genes: *SELL—*5’TACTGCATTCTCTGGGTTGG3’ and 5’CACCAAGGGCGATTTAATATG3’ (200 nM); *ICAM1—*5’AGCTTCTCCTGCTCTGCAAC3’ and 5’CATTGGAGTCTGCTGGGAAT3’ (100 nM) and *ITGAL—*5’GTTGACGTGGTGTATGAGAAGC3’ and 5’GTTGACGTGGTGTATGAGAAGC3’ (200 nM). The efficiency curve was performed with a cDNA1:10 dilution at an initial concentration of 50 ng/µL. We also used four reference genes (*ACTB—*5’GTCTTCCCCTCCATCGTG3’ and 5’CCACATAGGAATCCTTCTGACC3’; *HPRT1—*5’CTTTGCTTTCCTTGGTCAGG3’ and 5’TTCGTGGGGTCCTTTTCAC3’; *RPL19—*5’AGACCCCAATGAGACCAATG3’ and 5’GGATGATCAGCCCATCTTTG3’ and; *GAPDH—*5’CAACGACCACTTTGTCAAGC3’ and 5’TTCCTCTTGTGCTCTTGCTG3’, all with 200 nM concentration) as normalizers for our samples. The analysis was performed using two software programs: BestKeeper^34^ and GeNorm^35^. Both software programs indicated that the *GAPDH* gene would be the gene with the least stability among the reference genes tested for our samples. Thus, we chose the *ACTB, HPRT1*, and *RPL19* genes as references for this research.

### Quantitative real-time PCR (qPCR)

Reactions were prepared with 5 µL of ItaQ Universal SYBR Green Supermix (Bio–Rad, California, USA) 2 × , 2.5 µL of cDNA (concentration = 5 ng/µL) and varying volumes of Milli-Q® water and Primers, according to the concentration established in the concentration tests, to reach the final reaction volume of 10 µL. Analysis was performed with a 7500RT-PCR system (Applied Biosystems, California, USA) according to the manufacturer’s instructions. Each sample was assayed in triplicate. The mRNA expression was calculated using the Pfaffl method^36^, and the expression levels were given in arbitrary units (AU).

### Statistical analysis

The statistical analysis was performed using GraphPad Prism software (version 7.00 for Windows, GraphPad Software, California, USA). The Mann–Whitney or Wilcoxon tests were used to compare continuous random variables between two groups, and the Kruskal–Wallis test was used to compare three or more groups whose variables did not have a normal distribution. Spearman’s coefficient was used to verify linear associations between variables. All tests were conducted at the 5% significance level.

## Results

### SELL

*SELL* mRNA expression (n = 157 cases, of which 77 were benign and 80 were malignant) was higher in malignant (0.85 ± 1.54 AU) than in benign thyroid nodules (0.54 ± 0.71 AU, *p* = 0.0027). *SELL* mRNA expression could differentiate PTC from FA (*p* = 0.0018) and FTC from FA (*p* = 0.0078). Also, *SELL* mRNA expression was significantly lower in Hürthle cell tumors (0.45 ± 0.63 AU vs 0.95 ± 1.46 AU, *p* < 0.0001), but we could not demonstrate any other association with clinical or pathological features (Table 1). *SELL* expression was not associated with any characteristic of tumor aggressiveness or patient outcome.

**Table 1:** *SELL, ICAM1* and *ITGAL* mRNA expression in 191 thyroid nodule patients and clinical features.

mRNA expression			SELL		ICAM1		ITGAL	
			Median ± IR (n)	*p*-value	Median ± IR (n)	*p*-value	Median ± IR (n)	*p*-value
Nodules	Goiter		0.71 ± 0.92 (40)	0.0057*	0.46 ± 0.90 (34)	0.0114*	0.56 ± 1.03 (38)	0.4471*
	FA		0.47 ± 0.72 (37)		0.53 ± 1.24 (36)		0.86 ± 1.38 (43)	
	PTC		0.94 ± 1.62 (62)		0.93 ± 1.22 (58)		0.90 ± 1.49 (62)	
	FTC		0.82 ± 2.38 (18)		1.03 ± 3.30 (19)		1.26 ± 1.07 (18)	
Characteristics	Sex	Female	0.73 ± 1.38 (126)	0.9813	0.59 ± 1.02 (122)	0.4319	0.82 ± 1.64 (129)	0.4687
		Male	0.69 ± 1.03 (31)		0.72 ± 0.88 (25)		1.05 ± 1.13 (32)	
	Age at diagnosis		47.61 ± 14.46 (157)	0.5260**	47.66 ± 14.12 (137)	0.8960**	47.61 ± 14.46 (160)	0.5260**
	Tumor size		2.85 ± 1.82 (157)	0.8440**	3.09 ± 2.16 (139)	0.7000**	2.86 ± 1.82 (156)	0.8440**
	Hürthle cells	Present	0.45 ± 0.63 (80)	** < 0.0001**	0.44 ± 0.82 (75)	** < 0.0001**	0.68 ± 1.05 (87)	0.1365
		Absent	0.95 ± 1.46 (71)		0.95 ± 1.60 (69)		1.07 ± 1.65 (71)	
	Lymphocytic infiltration	Present	1.40 ± 2.16 (27)	0.1883	1.16 ± 3.04 (31)	**0.0064**	1.17 ± 1.54 (29)	**0.0244**
		Absent	0.88 ± 1.44 (32)		0.52 ± 0.96 (28)		0.49 ± 1.39 (33)	
	Extrathyroidal extension	Present	0.64 ± 1.50 (25)	0.0973	0.95 ± 1.12 (27)	0.5014	0.72 ± 0.95 (26)	**0.0111**
		Absent	1.02 ± 1.68 (47)		1.14 ± 3.22 (48)		1.25 ± 1.86 (47)	
	Invasion	Present	0.93 ± 1.42 (54)	0.8701	1.03 ± 1.79 (56)	0.5113	0.93 ± 1.42 (54)	0.8701
		Absent	0.85 ± 1.92 (17)		0.68 ± 2.33 (17)		0.85 ± 1.92 (17)	
	Lymphovascular infiltration	Present	0.72 ± 1.42 (34)	0.5223	1.78 ± 1.55 (36)	0.8910	0.74 ± 1.01 (35)	**0.0427**
		Absent	1.00 ± 1.60 (36)		1.07 ± 3.27 (38)		1.26 ± 1.96 (36)	
	Lymph node metastasis at diagnosis***	Present	0.88 ± 1.47 (34)	0.7642	0.73 ± 0.75 (33)	0.9232	0.75 ± 1.14 (34)	0.1603
		Absent	0.95 ± 1.97 (18)		1.11 ± 2.60 (13)		1.17 ± 1.83 (18)	
	Distant metastasis at diagnosis	Present	1.05 ± 1.74 (09)	0.4995	1.49 ± 3.20 (07)	0.3608	1.53 ± 2.18 (08)	**0.0217**
		Absent	0.72 ± 1.54 (42)		0.99 ± 1.30 (42)		0.57 ± 1.10 (43)	
	TNM	I + II	0.79 ± 1.60 (34)	0.2625	1.11 ± 2.18 (25)	0.7030	0.74 ± 1.67 (34)	0.7138
		III + IV	1.02 ± 2.23 (19)		0.84 ± 1.46 (19)		0.96 ± 1.64 (18)	

Mann–Whitney test; *Kruskal–Wallis test; **Spearman correlation.

***Lymph node metastasis at diagnosis analysis only considered PTC cases.

*IR* Interquartile Range; *FA* Follicular Adenoma; *PTC* Papillary thyroid cancer; *FTC* Follicular thyroid cancer.

Significant values are in [bold].

**Figure 1:**
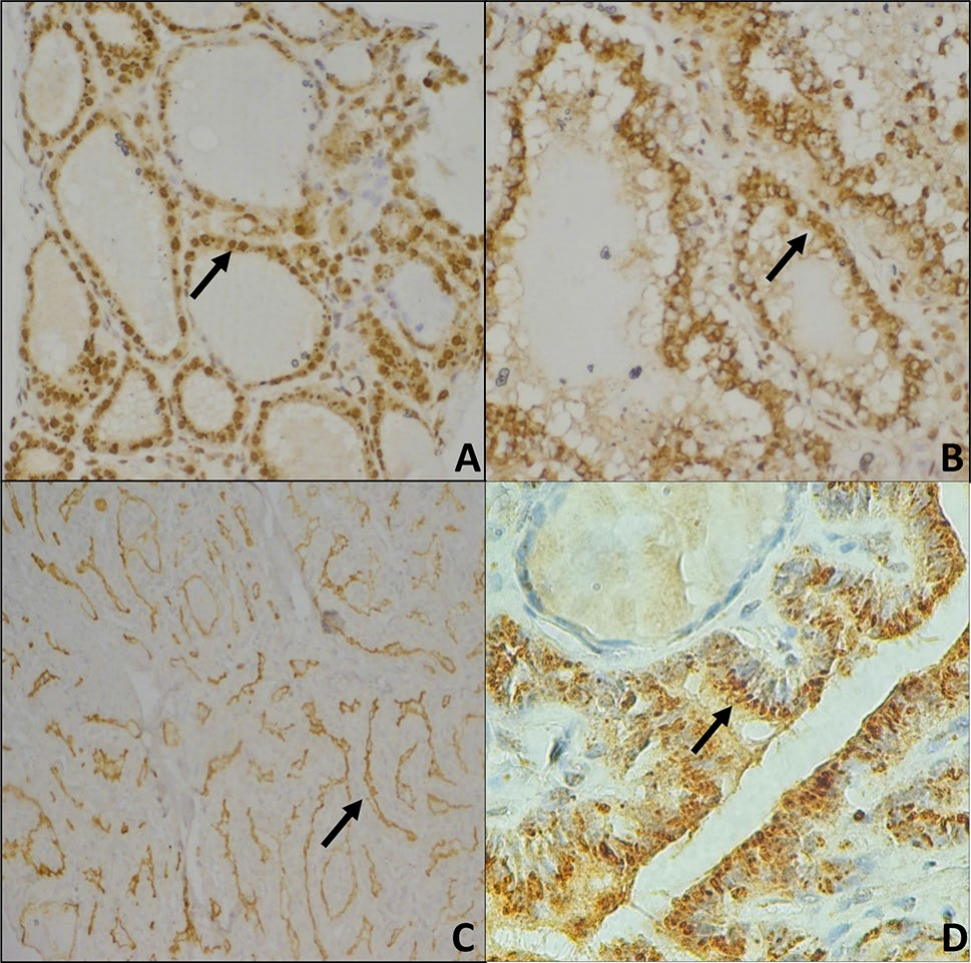
Protein expression evaluated from immunoistochemistry reaction. (**A**) Nuclear expression of L-selectin (20x); (**B**) Cytoplasmatic expression of L-selectin (20x); (**C**) Cytoplasmic Apical expression of ICAM-1 (10x) and; (**D**) Cytoplasmic expression of CD11a (100x).

Regarding protein expression (n = 229 cases, of which 98 benign and 131 malignant), nuclear expression (Fig. 1A) showed weaker intensity in malignant tumors (*p* = 0.0020) but not cytoplasm (*p* = 0.3344, Fig. 1B). However, both nuclear and cytoplasmic staining could differentiate follicular patterned lesions (Nuclear: PTC vs goiter, *p* < 0.0001; PTC vs FA, *p* = 0.0020; PTC vs FTC, *p* = 0.0133. FTC vs goiter, *p* < 0.0001; FTC vs FA, *p* < 0.0001, and Cytoplasmic: FA vs PTC, *p* = 0.0026; FA vs FTC, *p* = 0.0005 and Goiter vs FTC, *p* = 0.0062).

The group of patients younger than 55 years that were classified as TNM I + II (n = 32) did not differ from the remaining patients, both concerning mRNA gene expression (0.790 ± 1.710 vs III + IV, n = 7, 0.910 ± 1.880; *p* = 0.8397) and protein expression (nuclear, *p* = 0.4304 and cytoplasmic, *p* = 0.8040).

The nuclear and cytoplasmic protein expression of L-selectin were not correlated with any other clinical feature or to patient outcome (Table 2).

**Table 2:** L-selectin, ICAM-1 and LFA-1 protein expression in 275 thyroid nodule patients and clinical features—% (N).

Protein expression			L-selectin								ICAM-1				LFA-1			
			Nuclear				Cytoplasmic				Cytoplasmic–Apical				Cytoplasmic–Apical			
			Negative	Positive		*p*-value	Negative	Positive		*p*-value	Negative	Positive		*p*-value	Negative	Positive		*p*-value
				Weak	Moderate			Weak	Moderate			Weak	Moderate			Weak	Moderate	
Nodules	Goiter		9.1% (04)	90.9% (40)		0.0002*	15.9% (07)	84.1% (37)		0.2582	47.5% (19)	52.5% (21)		0.0962*	15.6% (07)	84.4% (38)		0.0489*
	FA		7.4% (04)	92.6% (50)			14.8% (08)	85.2% (46)			48.8% (21)	51.2% (22)			14.8% (08)	85.2% (46)		
	PTC		32.1% (21)	67.9% (89)			7.0% (08)	93.0% (100)			39.8% (47)	60.2% (71)			26.2% (27)	73.8% (76)		
	FTC		47.6% (10)	52.4% (11)			5.0% (01)	95.0% (18)			68.2% (15)	31.8% (7)			42.1% (08)	57.9% (11)		
Characteristics	Sex	Female	79.5% (31)	74.4% (61)	78.7% (85)	0.7323	72.0% (18)	79.8% (79)	76.0% (79)	0.6524	77.5% (79)	80.5% (66)	82.1% (32)	0.7932	74.0% (37)	82.4% (122)	68.2% (15)	0.1858
		Male	20.5% (08)	25.6% (21)	21.3% (23)		28.0% (07)	20.2% (20)	24.0% (25)		22.5% (23)	19.5% (16)	17.9% (07)		26.0% (13)	17.6% (26)	31.8% (07)	
	Age at diagnosis		46.5 ± 16.0	48.2 ± 14.6	51.1 ± 13.2	0.1642*	51.2 ± 12.3	48.9 ± 14.6	49.1 ± 14.6	0.7489**	50.6 ± 14.6	47.0 ± 13.0	45.6 ± 13.7	0.0897**	49.0 ± 14.3	46.2 ± 14.2	53.8 ± 8.6	0.0376**
	Tumor size		3.8 ± 2.9	2.9 ± 2.3	2.8 ± 2.0	0.2362*	3.1 ± 2.2	3.2 ± 2.5	2.8 ± 2.1	0.5384**	3.2 ± 2.4	2.4 ± 1.8	2.5 ± 1.7	0.0443**	3.2 ± 2.9	2.4 ± 1.5	3.0 ± 2.2	0.6017**
	Hürthle cells	Present	43.6% (17)	53.7% (44)	54.6% (59)	0.4769	84.0% (21)	69.6% (48)	48.1% (50)	**0.0034**	53.9% (21)	54.4% (56)	47.6% (39)	0.6288	50.0% (25)	62.4% (93)	40.9% (09)	0.0780
		Absent	56.4% (22)	46.3% (38)	45.4% (49)		16.0% (04)	30.4% (51)	51.9% (54)		46.2% (18)	45.6% (47)	52.4% (43)		50.0% (25)	37.6% (56)	59.1% (13)	
	Lymphocytic infiltration	Present	52.0% (13)	27.7% (13)	33.3% (09)	0.2000	66.7% (04)	42.6% (26)	25.0% (09)	0.0730	35.0% (11)	45.6% (21)	47.1% (16)	0.6579	41.4% (12)	50.0% (23)	50.0% (04)	0.7547*
		Absent	48.0% (12)	72.3% (34)	66.7% (18)		33.3% (02)	57.4% (35)	75.0% (27)		65.0% (13)	54.4% (25)	52.9% (18)		58.6% (17)	50.0% (23)	50.0% (04)	
	Extrathyroidal extension	Present	36.0% (09)	45.3% (24)	40.5% (15)	0.7283	25.0% (02)	39.1% (25)	47.6% (20)	0.4271	35.5% (11)	32.7% (17)	38.5% (15)	0.8496	46.4% (13)	28.8% (19)	30.0% (03)	0.2458
		Absent	64.0% (16)	54.7% (29)	59.5% (22)		75.0% (06)	60.9% (39)	52.4% (22)		64.5% (20)	67.3% (35)	61.5% (24)		53.6% (15)	71.2% (47)	70.0% (07)	
	Invasion	Present	87.5% (21)	69.2% (36)	65.7% (23)	0.1534	71.4% (05)	73.0% (46)	70.0% (28)	0.9462	64.5% (10)	70.6% (36)	59.5% (22)	0.5500	72.4% (21)	68.8% (44)	40.0% (04)	0.1519
		Absent	12.5% (03)	30.8% (16)	34.3% (12)		28.6% (02)	27.0% (17)	30.0% (12)		35.5% (11)	29.4% (15)	40.5% (15)		27.6% (08)	31.2% (20)	60.0% (06)	
	Lymphovascular infiltration	Present	44.0% (11)	37.7% (20)	65.7% (12)	0.6996	62.5% (05)	38.1% (24)	31.0% (13)	0.2327	28.8% (15)	39.5% (15)	35.5% (11)	0.5614	20.0% (02)	35.4% (23)	28.6% (08)	0.5615
		Absent	56.0% (14)	62.3% (33)	34.3% (24)		37.5% (03)	61.9% (39)	69.0% (29)		71.2% (37)	60.5% (23)	64.5% (20)		80.0% (08)	64.6% (42)	71.4% (20)	
	Lymph node metastasis at diagnosis***	Present	35.7% (05)	65.9% (27)	62.5% (20)	0.1288	40.0% (02)	57.8% (26)	66.7% (24)	0.4513	48.6% (17)	57.1% (20)	65.0% (13)	0.4844	47.6% (10)	56.5% (26)	75.0% (06)	0.4115
		Absent	64.3% (09)	34.1% (14)	37.5% (12)		60.0% (03)	42.2% (19)	33.3% (12)		51.4% (18)	42.9% (15)	35.0% (07)		52.4% (11)	43.5% (20)	25.0% (02)	
	Distant metastasis at diagnosis	Present	18.2% (04)	16.7% (07)	17.4% (04)	0.9882	40.0% (02)	16.7% (09)	14.3% (04)	0.3681	8.1% (03)	4.0% (01)	6.5% (01)	0.8124*	-	12.5% (04)	4.3% (01)	0.4115*
		Absent	81.8% (18)	83.3% (35)	82.6% (19)		60.0% (03)	83.3% (45)	85.7% (24)		91.9% (34)	96.0% (24)	93.3% (14)		100% (06)	87.5% (28)	95.7% (22)	
	TNM	I + II	5.0% (02)	72.5% (29)	22.5% (09)	0.0839	4.0% (02)	68.0% (34)	28.0% (14)	0.3214	53.10% (26)	26.50% (13)	20.40% (10)	0.5052*	2.4% (01)	61.0% (25)	36.6% (15)	0,0077*
		III + IV	9.7% (03)	45.2% (14)	45.2% (14)		7.9% (03)	52.6% (20)	39.5% (15)		42.90% (12)	39.30% (11)	17.90% (05)		25.0% (05)	30.0% (06)	45.0% (09)	

Chi-square test; *Fisher's exact test; **Mann–Whitney test, ***Lymph node metastasis at diagnosis was restricted to PTC cases.

*FA* Follicular Adenoma; *PTC* Papillary thyroid cancer; *FTC* Follicular thyroid cancer.

Significant values are in [bold].

### ICAM1

*ICAM1* mRNA expression (n = 147 cases, of which 70 were benign and 77 were malignant) was higher in malignant (0.99 ± 1.413 AU) than in benign thyroid nodules (0.46 ± 0.85 AU, *p* = 0.0001), and it differentiated PTC from goiter (*p* = 0.0030). Likewise *SELL*, *ICAM1* expression was lower in Hürthle cell tumors (0.44 ± 0.82 AU vs 0.95 ± 1.60 AU, *p* < 0.0001) and was higher in the presence of lymphocytic infiltration (1.16 ± 3.04 AU vs 0.52 ± 0.96 AU, *p* = 0.0064), but we could not associate it with any other clinical or pathological features (Table 1). *ICAM1* expression was also not associated with any characteristic of tumor aggressiveness or patient outcome.

Apical protein expression of ICAM-1 (Fig. 1C) was positive in 52.5% (n = 21) of our goiter samples, 51.2% (n = 22) of FA samples, and 31.8% (n = 7) of FTC samples. A moderate intensity of ICAM-1 distinguished malignant (25%) from benign nodules (6.0%; *p* = 0.0014). The group of patients younger than 55 years that were classified as TNM I + II (n = 34) did not differ from the remaining patients, both concerning mRNA gene expression (1.130 ± 3.461 vs III + IV, n = 7, 1.640 ± 3.540; *p* = 0.8722) and protein expression (*p* = 0.6766).

ICAM-1 expression correlated with smaller tumor size (*p* = 0.0443) but not with any other clinical feature or to the patients’ outcome (Table 2).

### ITGAL

**Figure 2:**
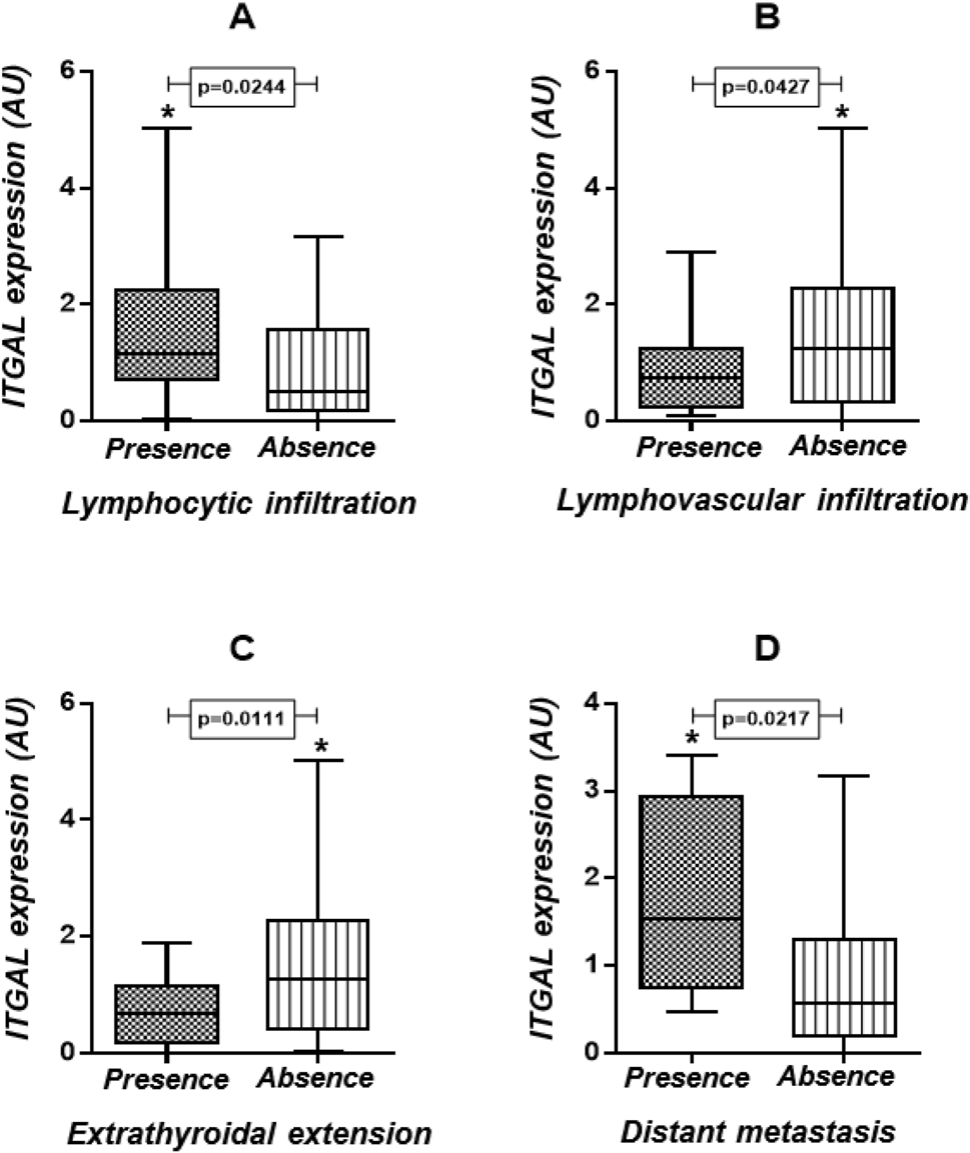
*ITGAL* mRNA expression correlations with clinical features. (**A**) Lymphocytic infiltration; (**B**) lymphovascular infiltration; (**C**) extrathyroidal extension and (**D**) distant metastasis at diagnosis. Mann–Whitney test.

*ITGAL* mRNA expression (n = 161 cases, of which 81 were benign and 80 were malignant) did not differ between malignant (1.04 ± 1.63 AU) and benign thyroid nodules (0.76 ± 1.21 AU, *p* = 0.2131) or among the histological types evaluated (PTC vs goiter, *p* = 0.1652; PTC vs FA, *p* = 0.7297; PTC vs FTC, *p* = 0.6662; FTC vs goiter, *p* = 0.2335; FTC vs FA, *p* = 0.4256 and FA vs goiter, *p* = 0.3029). However, *ITGAL* expression was correlated with several clinical pathological features associated with good prognosis: mRNA expression was higher in the presence of lymphocytic infiltration (1.17 ± 1.54 AU vs 0.49 ± 1.39 AU, *p* = 0.0244, Fig. 2A); in the absence of lymphovascular infiltration (1.26 ± 1.96 AU vs 0.74 ± 1.01 AU, *p* = 0.0427, Fig. 2B); and in the absence of extrathyroidal extension (1.25 ± 1.86 AU vs 0.72 ± 0.95 AU, *p* = 0.0111, Fig. 2C). Conversely, *ITGAL* was higher in cases with distant metastasis at diagnosis (1.53 ± 2.18 AU vs 0.57 ± 1.10 AU, *p* = 0.0217, Fig. 2D). We could not associate *ITGAL* expression with patient outcome (Table 1).

LFA-1 protein expression (Fig. 1D, n = 221 cases, of which 99 were benign and 122 were malignant) was more frequently negative in malignant tumors (*p* = 0.0168). We observed that this negativity was increasingly present in dedifferentiated samples (15,6% (n = 7) goiter; 14,8% (n = 8) FA; 26,2% (n = 26) PTC and 42,1% (n = 8) FTC were negative, *p* = 0.0489). Also, LFA-1 expression was more intense at stage III and IV(*p* = 0.0077). However, a comparison of patients younger than 55 years that were classified as TNM I + II (n = 31) with the remaining patients did not show any difference, both concerning mRNA gene expression (0.720 ± 1.550 vs III + IV, n = 7, 0.830 ± 2.230; *p* = 0.6112) and protein expression (*p* = 0.5519). Moderate expression of LFA-1 correlated with older age at diagnosis (*p* = 0.0376) but not with any other clinical feature or to the patients’ outcome (Table 2).

## Discussion

The importance of the immune response in tumor progression and patient prognosis is becoming increasingly clear, and our group is one of those who has been dedicating intense attention to the clinical application of the several cells and immune system mediators involved in this response^37–44^. However, interpreting data is difficult since tumor microenvironment elements can play a dual role, either suppressing malignancy or cooperating with tumor growth^45,46^. CAMs are essential for the migration of leukocytes to sites of inflammation and play an important role in stimulating intracytoplasmic signals that regulate cell differentiation, survival, and proliferation^47^. In fact, our data indicate that both *ICAM1* and *ITGAL* are associated with lymphocytic chronic infiltration. Due to their multiple interactions in different signaling pathways, CAMs correlate with a good prognosis in some tumor types, whereas in other neoplasm types, their expression is correlated with a worse prognosis and aggressive characteristics^5,7–9,13–16,20,21,26,27,48–52^. Taking advantage of a very well-characterized group of DTC patients carefully followed-up for more than 6 years by the same group in a single institution, we investigated the role of *SELL, ITGAL*, and *ICAM1* mRNA and protein expression in the characterization of thyroid nodules and their possible clinical utility in identifying aggressive tumors.

L-selectin is a molecule that acts mainly in the capture and rolling of migratory cells, both leukocytes and metastatic cells^10,49^. Its expression has been studied in several types of cancer, such as ovarian^50^, bladder^48^, and thyroid cancer^7,9,11^, and is usually related to tumor aggressiveness. *Muzza *et al.^9^ found higher expression of L-selectin in 15 PTC than in 4 normal (contralateral) thyroid tissues. We also observed higher *SELL* mRNA expression in malignant tumors than in benign thyroid nodules (*p* = 0.0027). We also showed that *SELL* mRNA expression could differentiate some of the histopathology follicular patterned cases, especially FA from FTC (*p* = 0.0078). Despite the trend toward higher *SELL* expression in tumors with lymphocytic infiltrate (1.40 ± 2.16 AU) when compared with tumors without this characteristic (0.88 ± 1.44 AU, *p* = 0.1883), we could not confirm the findings of *Muzza *et al.^9^ on this association^9^.

We observed decreased expression of both nuclear and cytoplasmic L-selectin in more aggressive histological types, suggesting that nuclear expression of this molecule decreases as neoplastic cells become less differentiated. On the other hand, Choudhary et al. observed that membrane L-selectin expression was higher in PTC than in benign nodules ^5^. This finding may be explained by L-selectin being cleaved after its activation, releasing its extracellular portion in soluble form^5,48^. Hence, the activation of this molecule may contribute to the cellular metastatic process. Indeed, the literature has reported a higher serum concentration of L-selectin in several types of malignant tumors^8,50,53,54^. The increase in soluble L-selectin and the possible relocation of its cytoplasmic portion may stimulate the increase in mRNA levels, explaining the higher *SELL* mRNA expression observed in malignant tumors alongside the lower expression of both nuclear and cytoplasmic proteins. However, note that L-selectin is a molecule participating in a process that involves a tangle of other molecules, cytokines, chemokines, transcription factors, signaling pathways, and other components that act in a systemic and integrated way. Thus, other yet undescribed factors may modulate L-selectin function.

ICAM-1 is a glycoprotein involved in both innate and adaptive immune responses^55^. It has been suggested that ICAM-1 acts as a facilitator of cancer cell spread through the recruitment of inflammatory cells that release stimulating factors for cell proliferation, angiogenesis, and invasion. This molecule also plays a role in the mechanisms of tumor cell progression, and studies have related it to the prognosis of some types of cancer^52,56^. Hayes and Seigel^15^ analyzed approximately 300 samples of normal, malignant, and metastatic tissues from various tumors, including thyroid cancer, and found overexpression of both the *ICAM1* gene and ICAM-1 protein in malignant tissues. More recently, Buitrago et al.^14^ confirmed higher expression of *ICAM1* in malignant tumors, corroborating our findings. However, despite also observing higher mRNA expression in the presence of lymphocytic infiltration, we could not associate ICAM1 with any characteristic of tumor aggressiveness or patient outcome.

LFA-1 is encoded by the *ITGAL* gene and is expressed in the leukocyte membrane^23^ but has also been described in several types of tumor cells^20,24–26^. LFA-1 has been associated with the progression of lymphomas^57^ and colorectal cancer^58^. *Papas *et al.^26^ evaluated LFA-1 expression in primary tumors and in metastatic tissues. They found a higher expression in primary tumors from patients without distant metastasis when compared to tumor tissue from patients who had no metastasis at diagnosis, proposing a negative correlation between the expression of LFA-1 and the metastatic process. The authors suggested a tumor suppressor role for LFA-1 in colorectal adenocarcinomas since its expression was higher in primary tumors from patients without distant metastasis. However, they also observed a higher expression of LFA-1 in the metastatic tissues when compared to the primary tumor, which may suggest a different role of LFA-1 after the initiation of the metastatic process since this molecule participates intensely in the migratory process. Our findings of lower *ITGAL* mRNA expression in cases with lymphovascular invasion (*p* = 0.0427) and extrathyroidal extension (*p* = 0.0111) and high mRNA expression in cases with lymphocytic infiltrate (*p* = 0.0244), reinforce a tumor suppressive role for LFA-1 in thyroid tumorigenesis. We know that the conformational structure of LFA-1 changes to interact with other CAMs, especially ICAM-1^18^ making us speculate that LFA-1 role depends on its conformational structure. Alternatively, it may be regulated by other signaling pathway molecules.

In conclusion, our data confirm an important role of CAMs in thyroid cell proliferation and tumor progression. In general, the protein expression of these molecules decreases as the cell dedifferentiation process occurs. However, the relatively long and positive course of most thyroid cancer patients, and the complex role of CAMs at the different stages of tumor evolution hinders the correlation of these molecules with tumor aggressiveness or patient outcomes in a clinical setting. On the other hand, we demonstrated that mRNA expression of *SELL* and *ICAM1* and protein expression of L-selectin and LFA-1 can help in the histological characterization of thyroid nodules with a follicular pattern.

## Data Availability

All data generated or analysed during this study are included in this published article.
